# Whole-genome sequencing and antimicrobial resistance in *Brucella melitensis* from a Norwegian perspective

**DOI:** 10.1038/s41598-018-26906-3

**Published:** 2018-06-04

**Authors:** Tone B. Johansen, Lonneke Scheffer, Veronica K. Jensen, Jon Bohlin, Siri L. Feruglio

**Affiliations:** 1 Division of Infection Control and Environmental Health, Norwegian Institute of Public Health, PO Box 4404 Nydalen, N-0403 Oslo, Norway; 2 Hanze University of Applied Sciences, Zernikeplein 7, 9747 AS Groningen, The Netherlands; 3 Present Address: Department of Informatics, University of Oslo, P.O. Box 1072 Blindern, 0316 Oslo, Norway

## Abstract

Brucellosis is a rarely encountered infection in Norway. The aim of this study was to explore all *Brucella melitensis* isolates collected in Norway from 1999 to 2016 in relation to origin of infection and antimicrobial resistance patterns. A total of 23 isolates were analysed by whole-genome sequencing and compared with selected sequences of *B. melitensis* available from NCBI. Additionally, SNP analysis in antibiotic resistance determining genes was performed. The majority belonged to the East Mediterranean clade (genotype II), while the remaining isolates belonged to the African clade (genotype III). These results indicate that human brucellosis in Norway is related to travels or migration from the Middle East, Asia or Africa, in accordance with results from Germany, Denmark and Sweden. Antibiotic susceptibility patterns were determined by broth microdilution method and/or gradient strip method. All isolates were susceptible for all tested antibiotics, except for rifampicin where phenotypical results indicated resistance or intermediate resistance in all isolates based on broth microdilution method, and in four isolates based on gradient strip testing. In contrast, screening of the *rpoB* gene did not reveal any mutations in the previously described *rpoB* “hot spot” regions related to rifampicin resistance, indicating overestimation of resistance based on phenotypical results.

## Introduction

Brucellosis is a worldwide zoonotic disease, with an estimated 500 000 new cases annually^1^. The disease is transmitted to humans by direct contact with infected animals or consumption of infected animal products, in particular unpasteurized milk products^2,3^. Several species of the genus *Brucella* can be pathogenic for humans, the most frequently encountered being *Brucella melitensis*^4,5^. The infection may present with an acute or insidious onset, with continued, intermittent or irregular febrile illness, fatigue, anorexia, weight loss, headache, arthralgia or generalized aching. The condition may progress to chronic disease with severe complications^5^. Brucellosis requires correct and protracted antimicrobial treatment^6,7^. Susceptibility testing is strongly recommended, as several studies have described resistance for commonly used antimicrobial drugs, such as rifampicin and trimethoprim/sulfamethoxazole^8–14^. However, resistance testing is not always performed, due to challenges concerning personnel biosafety. *Brucella* spp. is easily aerosolized and poses a high risk of laboratory acquired infections. A specialized BSL3 laboratory is therefore necessary^5,15,16^. In Norway, treatment of brucellosis follows WHO guidelines^5^ and includes a combination of doxycycline and gentamicin or alternatively doxycycline and rifampicin for children over eight years and adults. For younger children and pregnant women, doxycycline is replaced by trimethoprim/sulfamethoxazole^17^. As no data on resistance profiles from *Brucella* isolates in Norway currently exist, a relevant question is: are current empirical guidelines for treatment adequate?

Bovine brucellosis was eradicated from Norway in 1953 and brucellosis in sheep, goats and pigs has never been detected^18^. The infection is sporadically detected in humans, with 0–4 cases reported annually the last 20 years^19^. Most of the patients have been infected abroad, and commonly brucellosis is detected in migrants or travellers returning from endemic risk areas. Brucellosis is considered to be a re-emerging disease^20^, and travelling and globalization give reason for increased awareness.

In non-endemic countries as Norway, it is important to assess the epidemiological background of rare imported diseases such as brucellosis. The purpose of this study was to trace the origin of *Brucella* spp. infecting Norwegian patients in the period 1999 to 2016 using WGS based methods and comparison with publicly available *B. melitensis* sequences. Molecular epidemiological studies provide information about genetic grounds and origin for bacterial isolates, but such trace-back studies can be challenging as the *Brucella* bacteria are genetically quite conserved^21^. Multilocus variable tandem-repeat typing analysis (MLVA) has been the method of choice for epidemiological fingerprinting of *Brucella* spp^15,22^. This method is limited to specifically targeted regions in the genome. Whole-genome sequencing (WGS) is a more powerful tool for accurate typing of *Brucella* spp., as the entire genome of the bacteria can be studied and thereby increase the discriminatory power^23^. In addition, we have assessed antimicrobial resistance mechanisms in all isolates. This is the first study in Norway where human brucellosis is studied in relation to geographical source and antimicrobial resistance pattern.

## Results

### Sequencing and phylogeny

**Figure 1:**
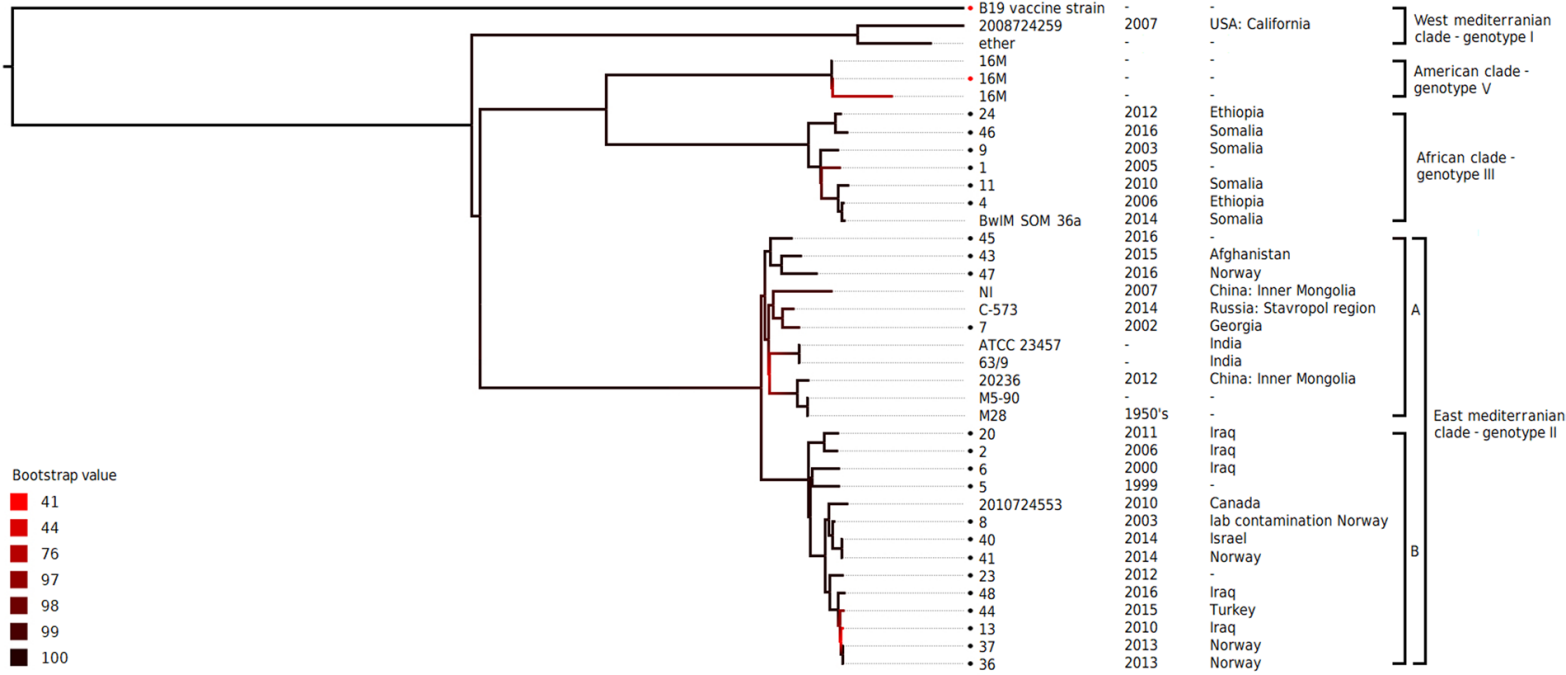
Whole-genome SNP-based phylogenetic tree illustrates origin of human brucellosis in Norway linked to Middle-East, Asia and Africa. A phylogenetic tree based on WGS analysis showing the relationship between 23 clinical isolates of *Brucella melitensis* isolated from patients in Norway compared to selected existing *B. melitensis* genomes available in the NCBI Genome Assembly and Annotation. Colours indicate bootstraps values. The Norwegian isolates are marked with dots. *B. abortus* vaccine strain B19 is included as outgroup.

In all, 23 clinical isolates and two reference strains (*B. melitensis* biovar 1 strain 16 M (ATCC 23456) and *B. abortus* B19 (vaccine strain)) were sequenced (Table 1). The average coverage depth and coverage breadth (nucleotide positions covered with at least 5 reads) are summarized in supplementary information. A phylogenetic tree was made to visualize the relationship between the different isolates of *B. melitensis* (Fig. 1). In addition, 13 previously published genome sequences of *B. melitensis* available from NCBI^24^ were included in this tree. The average genome coverage following alignment using Parsnp was 96.8% (individual values varying between 96.4% and 96.9%). RAxML was used to construct the phylogenetic tree from this alignment. Bootstrap values were included to show the strength of the analysis, and for most branches the bootstrap value was greater than 95/100, showing robustness. Two bifurcations in the tree have a bootstrap quality below 50/100. The *B. melitensis* 16 M reference sequence (isolate #22) grouped together with known 16 M genomes, as expected, and *B. abortus* B19 vaccine strain (#21) is the obvious outgroup compared to the other isolates.

**Table 1:** *Brucella melitensis* isolates.

Isolate #	Year	Sex	Age	Country of infection	Genotype
1	2005			nd	III
2	2006	F	33	Iraq	II
4	2006	F	34	Ethiopia	III
5	1999			nd	II
6	2000	F	38	Iraq	II
7	2002	M	22	Georgia	II
8	2003	M	54	Norway (lab infection)	II
9	2003	M	3	Somalia	III
11	2010	M	27	Somalia	III
13	2010	M	38	Iraq	II
20	2011	F	53	Iraq	II
21	2010			*B. abortus* B19	
22	2010			*B. melitensis* 16 M	V
23	2012	M	50	nd	II
24	2012			Ethiopia	III
36	2013	M	43	Norway	II
37	2013	F	22	Norway	II
40	2014	M	68	Israel	II
41	2014	M	47	Norway	II
43	2015	M	15	Afghanistan	II
44	2015	M	14	Turkey	II
45	2016	M	59	nd	II
46	2016	M	39	Somalia	III
47	2016	M	50	Norway	II
48	2016			Iraq	II

Genotype determined by WGS. Genotype II resembles East Mediterranean clade, genotype III resembles African clade and genotype V resembles American clade.

WGS gave a good resolution, separating all isolates but two; isolates #36 and #37 (Fig. 1). There is virtually no distance between them in the phylogenetic tree, indicating a common source. The isolates originated from family members that developed brucellosis following a common travel, which explains the similarity. Isolates #40 and #41 are also closely related in the tree, and one might assume a common source. However, epidemiological data showed two different countries of origin, Israel and Norway. As infection in Norway is not likely, this discrepancy might be due to incorrect collection of epidemiological data.

When compared with selected isolates of *B. melitensis* available from NCBI^24^, the isolates from our dataset clustered in two previously described lineages; the East Mediterranean (EM) clade (genotype II) and the African (Af) clade (genotype III)^23,25^. Isolates from patients infected in Iraq, Israel, Turkey, Afghanistan, Georgia and Norway clustered in the EM clade^25^. One of these isolates (# 8) was reported to the Norwegian Surveillance System for Communicable Diseases^19^ as a case of laboratory infection. Unfortunately, the original isolate was not available for comparative analysis. The isolates in the EM clade further divided into two sub-clusters (A and B) (Fig. 1). In sub-cluster A, published genomes from Asia (China, India and Russia)^26–30^ clustered together with analysed isolates originating from; a patient infected in Afghanistan, one in Georgia, one with unknown country of infection and one reported as infected in Norway. Sub-cluster B included isolates from patients infected in Iraq, Turkey, Israel and Norway together with the published genome of a isolate originating from a patient from Syria (NCBI 2010724553)^31^.

The sequenced isolates from the two patients reported as infected in Ethiopia and the three patients infected in Somalia clustered in the African (Af) clade (genotype III) (Fig. 1). Additionally, one sequence originated from a patient with unknown country of infection. A published genome from an isolate originating from Somalia representing this clade in a previous publication (BwIM_SOM-36a)^23^, clustered closely together with the isolates from this dataset. Isolates matching the West Mediterranean clade (genotype I), the European clade (genotype IV) or the American clade (genotype V)^23,25^, were not detected in our dataset.

### Antimicrobial susceptibility

The obtained MIC values for all tested antibiotics are shown in Tables 2 and 3. All isolates were susceptible for doxycycline, streptomycin and gentamicin. The obtained MIC values for imipenem, chloramphenicol and amoxicillin clavulanic acid are described in Tables 2 and 3. By broth microdilution method, 17 out of the 23 isolates (74%) were intermediate resistant for rifampicin (MIC = 2 µg/mL) and 6 isolates (26%) were resistant (MIC = 4 µg/mL). In parallel, isolates were also tested by gradient strip method for rifampicin^10,14,32^, and 4 isolates (17%) were intermediate resistant (MIC = 2 µg/mL) while the remaining 19 isolates (83%) were susceptible (MIC = 0.25–1 µg/mL). All isolates were susceptible for trimethoprim-sulfamethoxazole by gradient strip testing, (MIC = 0.032–0.064 µg/mL).

**Table 2:** Results of AST testing of *Brucella melitensis*.

Antibiotic agent	CLSI Breakpoints for *Brucella* spp. (µg/mL)^40^				
	Mode (µg/mL)	Range (µg/mL)	S I R ≤ = ≥		
Rifampicin (MD)^a^	2	2–4	1	2	4
Rifampicin (GS)^a^	1	0.5–2	1	2	4
Doxycycline (MD)	0.125	0.063–1	1	—	—
Streptomycin (MD)	4	2–4	16^b^	—	—
Gentamicin (MD)	1	1–2	4	—	—
Imipenem (MD)	2	2–4	nd	nd	nd
Chloramphenicol (MD)	4	2–8	nd	nd	nd
Amoxicillin Clavulanic acid (MD)	<1/0.5	<1/0.5–2/1	nd	nd	nd
Trimethoprim- Sulfamethoxazole (GS)	0.064^c^	0.032–0.064^c^	2/38	—	—

Method used: MD = Microdilution method, (GS = Gradient strip method. ^a^CLSI interpretation of *Haemophilus influenza* (fastidious bacteria). ^b^For incubation conditions with 5% CO_2_. ^c^Trimethoprim-Sulfamethoxazole; Only the trimethoprim portion of the 1/19 drug ratio is displayed. nd = not determined.

**Table 3:** Distribution of MIC values among *Brucella melitensis* isolates tested.

MIC (µg/mL)	Broth dilution method No. of isolates							Gradient strip method No. of isolates	
	RIF	DOX	STR	GEN	IMP	CMP	AMC	T/S	RIF
0.016									
0.032								10	
0.064		4						13	
0.125		7							
0.25		5							
0.5		6							7
1		1		19			18^a^		12
2	17		2	4	21	4	5^b^		4
4	6		21		2	13			
8						6			
16									
32									
Total	23	23	23	23	23	23	23	23	23

^a^MIC ≤ 1/0,5. ^b^MIC = 2/1. RIF = rifampicin, DOX = doxycycline, STR = streptomycin, GEN = gentamycin, IMP = Imipenem, CMP = Chloramphenicol, AMC = Amoxicillin Clavulanic acid, T/S = Trimethoprim-Sulfamethoxazole.

### SNP analysis in antibiotic resistance determining genes

The gene and protein sequences for seven known antibiotic resistance-associated genes (*rpoB, folA*, *folP*, g*yrA*, *gyrB*, *parC*, *parE*)^33^ were analysed for SNP variants comparing the sequenced isolates to the reference strain *B. melitensis* 16 M (#22) (Table 4). Three different SNP variants were detected in *rpoB*, a gene associated with rifampicin resistance^33,34^. The mutations detected in the *rpoB* gene in our data were located at nucleic acid position 1174 [392-Glu (GAG)◊Asp (GAC)], 1185 [629-Ala (GCG)◊Val (GTG)] and 2953 [985-Ala [GCC)◊Val (GTC)]. These alterations are different from mutations previously described as a cause of rifampicin resistance in *Brucella*^34^. Additionally, the SNP alterations were not restricted to the four isolates phenotypically intermediate resistant to rifampicin based on gradient strip testing (#43, 45, 46 and 47). The SNP changes therefore does not seem associated with rifampicin resistance. The observed SNP variants were however observed to be specific for certain lineages and sub-clusters based on WGS analysis; the SNP in position 1174 was detected in two related isolates within the Af clade (#24 and #46), SNP in position 1185 was common for all isolates in the EM clade, and the SNP in position 2953 was restricted to the isolates in sub-cluster A in the EM clade (Fig. 1, Table 4).

**Table 4:** Mutations detected in known antibiotic resistance determining genes relative to *B. melitensis* 16 M^33^.

Gene; encoding (antibiotic)	DNA position	Amino acid/codon change	Clade; Isolate no.
*rpoB*; DNA dependent RNA polymerase subunit beta (rifampicin)	1174	392-Glu (GAG)◊Asp (GAC)	Af; 24,46
	1185	629-Ala (GCG)◊Val (GTG)	EM; 2,5,6,7,8,13,20, 23,36,37,40,41,43,44,45,47,48
	2953	985-Ala (GCC)◊Val (GTC)	EM (subcluster A); 7,43,45,47
*folA*; Dihydrofolate reductase (trimetoprim)	73	217-Arg (CGG)◊Leu (CTG)	Af; 1,4,9,11,24,46
*folP*; Dihydrofolate synthase (sulfamethoxazole)	631	211-Phe (TTC)◊Leu (CTC)	EM; 2,5,6,7,8,13,20, 23,36,37,40,41,43,44,45,47,48
*gyrA*; DNA gyrase subunit A (fluoroquinolone)	1759	599-Leu (CTG)◊Val (GTG)	EM; 2,5,6,7,8,13,20,23, 36,37,40,41,43,44,45,47,48 + Af; 1
*gyrB*; DNA gyrase subunit B (fluoroquinolone)	703	235-Thr (ACC)◊Ile (ATC)	21*
*parC*; DNA topoisomerase 4 subunit A (fluoroquinolone)	799	267-Arg (CGC)◊His (CAC)	Af; 1,11,4,9
	1318	440-Ala (GCT)◊Val (GTT)	21*
	1693	565-Phe (TTC)◊Leu (TTG)	EM; 44
	2164	722-Thr (ACC)◊Ser (AGC)	21*
*parE*; DNA topoisomerase 4 subunit B (fluoroquinolone)	27	79-Asn (AAC)◊Ser (AGC)	Af; 1,4,9,11,24,46

Baseline = *B. melitensis* 16 M (dataset 22).

Af = African clade, EM = East Mediterranean clade.

*21 is the reference strain *B. abortus* vaccine strain B19.

One mutation was detected in *folA*, a structural gene coding for dihydrofolate reductase and described to be involved in resistance mechanisms for trimethoprim^33^. The detected SNP at position 73 [217-Arg (CGG)◊Leu (CTG)] was present in all isolates belonging to the Af clade in the current dataset. In *folP*, the gene coding for dihydrofolate synthase and associated with sulfamethoxazole resistance^33^, one SNP difference was detected compared to the reference strain at position 631 [211-Phe (TTC)◊Leu (CTC)]. This SNP was detected in all isolates in the EM clade. However, as no T/S resistance was detected by phenotypical analysis, the relevance of these findings is uncertain.

Changes in the genes *gyrA*, *gyrB*, *parC* and *parE* were also detected (Table 4). These genes are all known as fluoroquinolone-resistance determining genes^33^, coding for DNA gyrase and DNA topoisomerase respectively^33^. The described mutation in *gyrA* did not correspond with mutations related to fluoroqinolone resistance described earlier^35^. The SNP detected in g*yrA* in position 1759 [599-Leu (CTG)◊Val (GTG)], was detected in all isolates in the EM clade, but was also detected in one isolate in the Af clade (#1). The mutation in the *gyrB* gene was detected only in the reference strain *B. abortus* B19 (#21), and not in any *B. melitensis* isolates. Four SNP differences were detected in *parC*, of which two was only present in *B. abortus* (#21), and one SNP difference was detected in *parE*. The SNP difference in *parE* in position 27 [79-Asn (AAC)◊Ser (AGC)] was present in all isolates in the Af clade, and the SNP at position 799 [267-Arg (CGC)◊His (CAC)] in *parC* was present in a sub-cluster in the Af clade. These SNP differences has to not been previously described.

## Discussion

This is the first investigation of brucellosis in Norwegian patients. We sequenced all clinical isolates collected between 1999 and 2016. The results provide valuable information on origin and transmission routes, and support the use of whole-genome sequencing for trace-back analyses of *B. melitensis*. Whole-genome sequencing is becoming a useful tool for molecular epidemiological studies of infectious diseases, providing better resolution compared to other techniques, like MLVA^23^. WGS provides the possibility to elucidate the geographical origin of an isolate or an outbreak, given no or unclear information on the probable country of infection for the patient. As the use of sequencing will increase, more genomes will be publicly available, adding further information about phylogeny and molecular epidemiology of brucellosis.

Our data demonstrates that human brucellosis in Norway is linked to travelling and migration from the Middle East, Asia or Africa, and our findings show that travel history and genetic epidemiological sequence data harmonize well. The majority of the isolates clustered in the East Mediterranean clade (genotype II). This is in accordance with results from Germany, Denmark and Sweden, and reflects similar travelling and migration patterns in northern Europe^15,23,36^. The East Mediterranean clade further divided into two sub-clusters (A and B), separating isolates with origin in the Middle-East from isolates originating from the Caucasus region and Central Asia. These findings are in line with data from other studies^23,25^. The isolates from patients infected in Ethiopia and Somalia clustered in the African clade (genotype III), as expected. Our findings resembles data based on MLVA analysis from Sweden^15^.

The five isolates reported from patients infected in Norway clustered in the East Mediterranean clade. One of them was a laboratory infection, while the origin of the remaining four isolates remains unclear. This might be due to clinical misreporting to the Norwegian Surveillance System for Communicable Diseases (MSIS)^19^, but infection from imported food cannot be ruled out. This raises the question if such sources, especially unpasteurized dairy products, might be a risk factor of consideration. Although the number of reported cases in Norway is low, the incidence of brucellosis in Northern Europe is increasing^23,36^, hence there is a reason to be alert and have an increased awareness about this disease.

In this study we have also analysed phenotypical resistance patterns and compared the results with WGS data, both to evaluate phenotypic test results and to investigate possible genetic markers that could predict resistance. Analysis of such genetic markers might provide a much needed alternative to phenotypic testing, simplifying the strict laboratory biosafety requirements necessary. To date, there are only few studies comparing phenotypical susceptibility testing with genetic sequencing results. However, these studies have demonstrated discrepancies between phenotypical *in vitro* susceptibility testing and sequencing^37–39^. Specific mutations in the gene coding for the β subunit of the DNA dependent RNA polymerase, *rpoB*, enable resistance for rifampicin in *Brucella* spp. as well as in other bacteria^33^. WGS data from our study did not reveal any mutations in the two previously described *rpoB* “hot spot” regions related to resistance^34^. Resistant or intermediate resistant phenotypes for rifampicin were however detected; by the broth microdilution method where all the 23 tested isolates showed resistant or intermediate resistant profiles, and in lesser extent by the gradient strip method where four isolates were reported as intermediate resistant. Our findings question if the recommended broth microdilution method by the Clinical and Laboratory Standards Institute^40^, and to some extent the gradient strip method, might overestimate *in vitro* rifampicin resistance in *B. melitensis*. This topic needs to be further addressed in larger multicentre studies.

Marianelli *et al*. has suggested that sequencing of the *rpoB* gene could be used a s a tool for genotyping and species determination of *Brucella* spp.^41^. Our results support this, as mutations in the *rpoB* gene were detected in several of our isolates. However, we could not find any correlation between these mutations and the phenotypical resistance test results. Noteworthy, the observed mutations distributed consistently within certain clades or sub-clusters in the dendrogram. Similar findings have been previously described (DNA position 1185 and 2953)^23,41,42^, while the SNP in DNA position 1174 from two isolates in the African clade is a novel finding.

In our cohort, all tested isolates were phenotypical susceptible to trimethoprim-sulfamethoxazole. By WGS analysis, one SNP change was detected in several isolates in *folP*, the gene related to sulfamethoxazole resistance. Additionally, six of the isolates had a mutation in the *folA* gene, previously described to be associated with trimethoprim resistance^33^. The SNP in *folP* was specific for the East Mediterranean clade, while the mutation in *folA* was detected in all isolates in the African clade. As these isolates were phenotypical susceptible, the association with resistance seems to be negligible, and the relevance of this finding needs to be further addressed.

Based on our results, occurrence of antibiotic resistance in *B. melitensis* is very low, and hence, current Norwegian empirical guidelines for treatment seem valid. Although the number of isolates in this study is limited, they originate from different continents and reflect a broad geographical area as well as a long time span. The results must be interpret with care, but our data question the reported challenge of rifampicin resistance described in some studies. There is a need for an updated and standardized methodology for resistance testing of *Brucella* spp., for establishing breakpoints for additional antibiotics and a need for more research into mechanisms determining resistance in *Brucella* spp. and possible genetic markers for resistance.

## Materials and Methods

### Study design and isolates

In this study, all available isolates from culture positive brucellosis patients registered in Norway in the period 1999 to 2016 were included. The Norwegian Institute of Public Health (NIPH) has the national reference function for *Brucella* spp. in Norway, and collects all the available clinical isolates. Patient data was obtained from the Norwegian Surveillance System for Communicable Diseases^19^. A total of 23 clinical isolates were included (Table 1). In addition, two reference strains, *B. melitensis* biovar 1 strain 16 M (ATCC 23456) and *B. abortus* B19 (vaccine strain), were sequenced and included for analysis. All procedures involving infectious agents were performed according to national regulations in a certified biosafety level 3 laboratory.

### Culture and identification

All isolates were cultured from freezing stock on Colombia blood agar plates for 48 hours at 37 °C in an enriched atmosphere before further processing. All isolates had previously been confirmed as *Brucella* spp. by real time PCR detecting the *Brucella* specific gene *bcsp31*^43^ and by MALDI-TOF MS analysis^44^. Isolates were confirmed as *Brucella melitensis* by Bruceladder multiplex PCR^45–47^. Inactivation was performed with NucliSENS® lysis buffer (Biomerioux, Durham, NC, USA).

### DNA isolation and whole-genome sequencing

Genomic DNA was extracted by QiaAmp DNA minikit (Qiagen, Hilden, Germany). Concentration of genomic DNA was quantified using Qubit® dsDNA BR Assay kit and the Qubit 2.0 Fluorometer (Thermo Fisher Scientific, Waltham, MA, USA). Illumina sequencing libraries were generated with the High Throughput Library Preparation Kit (KAPA Biosystems, Wilmington, Massachusetts, USA) following the manufacturer’s protocol. Individual libraries were indexed with NEXTflex barcodes (Bio [SIC] Scientific, Austin, Texas, USA). Sequencing was performed using an Illumina MiSeq platform with 2 × 150-bp paired-end reads (Illumina, San Diego, CA, USA)^48^.

### Genome assembly and analysis

Before assembly, the sequence data was trimmed using Trimmomatic version 0.35 to remove the adapter sequences and trim away low quality ends^49^. The trimmed sequence reads were then mapped to a reference genome using BWA-MEM (version 0.7.15-r1140)^50^. *B. melitensis* biovar 1 strain 16 M with chromosome I (NC_003317) and chromosome 2 (NC_003318) (NCBI Genbank®) was used as reference genome for the mapping. The output of this reference based assembly was processed using SAMtools (version 1.3.1, using htslib 1.3.1), BCFtools (version 1.2, using htslib 1.2.1)^51^ and Picard Tools (version 2.7.0) (http://broadinstitute.github.io/picard/). First, read mate errors were fixed using SAMtools fixmate, and duplicate reads were marked with Picard Tools. A pileup file was made using SAMtools mpileup with BAQ-recalculation enabled, to avoid indel artifacts. Using BCFtools, the variants (SNPs and indels) were extracted from the pileup file and consensus sequences were created for each of the 23 sequenced isolates and for the two reference strains. The average coverage depth and coverage breadth (nucleotide positions covered with at least 5 reads) were calculated using SAMtools.

These consensus sequences were annotated using Prokka (version 1.11)^52^. The gene and protein sequences for seven antibiotic resistance-associated genes (*folA*, *folP*, *gyrA*, *gyrB*, *parC*, *parE*, *rpoB*) were extracted from the GenBank files created by Prokka to explore possible genetic alterations linked to antibiotic resistance and compared to *B. melitensis* 16 M and other published genomes.

### Phylogenetic analysis

A phylogenetic tree was created using the 25 consensus sequences resulting from the reference-based assembly, including the sequenced genomes for the reference strains *B. melitensis* 16 M and *B. abortus* vaccine strain B19. In addition, selected existing *B. melitensis* genomes available in the NCBI Genome Assembly and Annotation report^24^ were included in the analysis. Assemblies with completion level ‘complete’ or ‘chromosome’ were selected. In total, 13 genomes were selected. All 38 genomes were aligned to each other using Parsnp (version 1.2) from the Harvest suite^53^. HarvestTools (version 1.2) was used to convert the Parsnp output to a multiple sequence alignment in FASTA format, which was then converted to Phylip format. The resulting multiple sequence alignment was used as the basis for creating a phylogenetic tree. To decide which substitution model to use when building the phylogenetic tree, the function phymltest of R package ‘ape’ was used. This function tests multiple substitution models (using PhyML) and calculates their Akaike Information Criterion (AIC). This showed that the most complex substitution model, GTR + Γ + I, resulted in the lowest AIC. The GTR + Γ + I model has thus been selected for building the phylogenetic tree. A maximum likelihood tree was built using RAxML (version 8.2.9)^54,55^ with the substitution model GTR + Γ + I. The Newick tree generated by Parsnp was used as the starting tree. To test the robustness of this tree, a bootstrap analysis was performed with replications set to 100.

### Antimicrobial susceptibility testing

All isolates were tested for antibiotic resistance to rifampicin (RIF), doxycycline (DOX), streptomycin (STR), gentamicin (GEN), imipenem (IMP), chloramphenicol (CMP) and amoxicillin/clavulanic acid (AMC) by broth microdilution testing according to Clinical and Laboratory Standards Institute (CLSI) guidelines^40^. The testing was performed in accordance with the working group for AST testing on highly pathogenic bacteria in the EU joint action “Efficient response to highly dangerous and emerging pathogens at EU level” (EMERGE) (http://www.emerge.rki.eu/Emerge/EN/Home/Homepage_node.html). All isolates were suspended in saline water to a 0.5 McF turbidity, and suspended in in *Brucella* broth adjusted to pH 7.1 ± 0.1 (BD Diagnostic Systems, Hunt Valley, MD, USA). Microdilution plates (Micronaut-AST IMB gramnegative MIC plates from Merlin Diagnostika GmbH, Berlin, Germany) were inoculated and incubated at 35 ± 2 °C with 5% CO2 for 48 h. Quality control assays were performed with *Escherichia coli* ATCC #25922 and *Streptococcus pneumoniae* ATCC #49619. Phenotypical resistance to fluoroquinolones was not evaluated in this study.

Rifampicin (RIF) and trimethoprim-sulfamethoxazole (T/S) resistance was additionally tested with gradient strip method. Susceptibility for T/S is only reported from gradient strip method, as testing in broth microdilution is known to generate false resistance due to high concentrations of thymidine in the *Brucella* broth^56^. Gradient strip method was performed using Liofilchem® MIC Test Strips (Liofilchem s. r. l., Roseto degli Abruzzi, Italy) as described elsewhere^10,14,32^. Briefly, a suspension of bacteria adjusted to 0.5 McF units was inoculated on Mueller-Hinton plates supplemented with 5% sheep blood and the gradient strips applied. The plates were incubated at 35 °C ± 2 °C with 5% CO_2_ for 48 h before reading. MIC values were interpret in accordance with CLSI guidelines for potential agents of bioterrorism^40^. The following breakpoints for susceptibilities were used: GEN ≤ 4, STR ≤ 16, DOX ≤ 1 and SXT ≤ 2/38. For RIF, CLSI-interpretation of *Haemophilus influenzae* (fastidious bacteria) was used; S ≤ 1, I = 2, R ≥ 4^40^. Susceptibility breakpoints for IMP, CMP and AMC have not been established for *Brucella* spp.

### Ethical approval

The study was performed in accordance with relevant guidelines and regulations and no experiments were performed on humans and/or human tissue samples. The study was approved by the Regional Committee for Medical Research Ethics in South Eastern Norway (REK Sør-Øst) (reference no 2016/2036).

### Accession codes

The genomes included in the present study are available in the European Nucleotide archive (ENA) database (accession no. PRJEB23353)^57^.

## Electronic supplementary material

Supplementary information
