# Supplementary material for: Whole-genome sequencing and antimicrobial resistance in Brucella melitensis from a Norwegian perspective
Source: Sci Rep. 2018 Jun 4;8:8538. doi: 10.1038/s41598-018-26906-3 (PMC5986768; doi:10.1038/s41598-018-26906-3)
Supplement: Supplementary file 1 — Supplementary information [file 41598_2018_26906_MOESM1_ESM.pdf]

Whole-genome sequencing and antimicrobial resistance in *Brucella melitensis* from a Norwegian perspective.

Tone B. Johansen[1,*], Lonneke Scheffer[1,2#], Veronica K. Jensen[1], Jon Bohlin[1,+], and Siri L. Feruglio[1,+]

[1]Division of Infection Control and Environmental Health, Norwegian Institute of Public Health, PO Box 4404 Nydalen, N-0403 Oslo, Norway

[2]Hanze University of Applied Sciences, Zernikeplein 7, 9747 AS Groningen, the Netherlands

* Correspondence should be addressed to T.B.J (email: Tone.Johansen@fhi.no)

[+] these authors contributed equally to this work.

#Present address: Department of Informatics, University of Oslo, P.O. Box 1072 Blindern, 0316 Oslo, Norway

Supplementary information

Supplementary information. Calculated average coverage depth and coverage breadth for *Brucella melitensis* isolates. Calculated using SAMtools.

Isolate no.	Average coverage depth	Coverage breadth (% covered by 5 or more reads)
1	40.10	99.91%
2	82.05	99.93%
4	19.66	99.88%
5	71.94	99.91%
6	36.75	99.91%
7	45.65	99.91%
8	62.25	99.92%
9	65.79	99.95%
11	56.44	99.95%
13	32.29	99.90%
20	25.10	99.88%
23	76.91	99.92%
24	64.83	99.95%
36	39.36	99.91%
37	20.25	99.83%
40	44.69	99.92%
41	83.69	99.92%
43	110.18	99.91%
44	48.81	99.91%
45	25.29	99.86%
46	48.33	99.90%
47	18.06	77.99%
48	70.25	99.92%
